# Improved computer-aided detection of pulmonary nodules via deep learning in the sinogram domain

**DOI:** 10.1186/s42492-019-0029-2

**Published:** 2019-11-22

**Authors:** Yongfeng Gao, Jiaxing Tan, Zhengrong Liang, Lihong Li, Yumei Huo

**Affiliations:** 1 Department of Radiology, State University of New York, Stony Brook, NY 11794 USA; 2 Departments of Computer Science, City University of New York/CSI, Staten Island, NY 10314 USA; 3 Engineering and Environmental Science, City University of New York/CSI, Staten Island,, NY 10314 USA

**Keywords:** Computer-aided detection, Computed tomography, Deep learning, Lung, Sinogram

## Abstract

Computer aided detection (CADe) of pulmonary nodules plays an important role in assisting radiologists’ diagnosis and alleviating interpretation burden for lung cancer. Current CADe systems, aiming at simulating radiologists’ examination procedure, are built upon computer tomography (CT) images with feature extraction for detection and diagnosis. Human visual perception in CT image is reconstructed from sinogram, which is the original raw data acquired from CT scanner. In this work, different from the conventional image based CADe system, we propose a novel sinogram based CADe system in which the full projection information is used to explore additional effective features of nodules in the sinogram domain. Facing the challenges of limited research in this concept and unknown effective features in the sinogram domain, we design a new CADe system that utilizes the self-learning power of the convolutional neural network to learn and extract effective features from sinogram. The proposed system was validated on 208 patient cases from the publicly available online Lung Image Database Consortium database, with each case having at least one juxtapleural nodule annotation. Experimental results demonstrated that our proposed method obtained a value of 0.91 of the area under the curve (AUC) of receiver operating characteristic based on sinogram alone, comparing to 0.89 based on CT image alone. Moreover, a combination of sinogram and CT image could further improve the value of AUC to 0.92. This study indicates that pulmonary nodule detection in the sinogram domain is feasible with deep learning.

## Introduction

According to American Cancer Society, lung cancer is by far the leading cause of cancer-related deaths in the United States. In 2019, there are about 228,150 new cases (116,440 in men and 111,710 in women) of lung cancer being diagnosed. An estimated 142,670 deaths (76,650 in men and 66,020 in women) from lung cancer will occur [1]. Currently, the reported 5-year survival rate for lung cancer is only 19%. Early detection of lung cancer is the key to prevent lung cancer and improve survival rate.

Computer aided detection (CADe) system has been developed as a second reader to help radiologists to efficiently locate and diagnose pulmonary nodules, thus reducing human interpretation burden. Traditional CADe systems for lung nodule detection are based on hand engineered features. Most commonly used engineered features include three types of features: intensity-based statistical features, geometric features, and gradient features [2]. Messay et al. [3] evaluated 245 of the above features. Among them, the geometric features are computed based on the shape and position information of lung nodules, whereas the intensity and gradient features are computed from computer tomography (CT) images using the boundaries defined by nodule candidates mask. Cascio et al. [4] proposed a stable 3-dimensional (3D) mass spring model, in which the directed contour information and shape knowledge have been utilized to automatically detect lung nodules. Choi et al. [5] introduced a novel 3D shape-based feature descriptor to detect pulmonary nodule candidates, which were further refined using an iterative wall elimination method. Han et al. [6] proposed a fast and adaptive CADe scheme for lung nodule detection, in which ten geometric or shape features, sixteen intensity features, fifteen gradient features, and eight Hessian eigenvalue based features are extracted for false positive (FP) reduction. Peña et al. [7] proposed a minimal characteristics extraction technique for lung nodule detection. After applying 3D blob algorithm associated with a connectivity algorithm to select the initial nodule candidates (INCs), they extracted eight minimal representative characteristics of the possible candidates for detection of lung nodules. Though hand engineered features have been proven very effective, studying and extracting those features are time consuming and expensive. It also requires expert knowledge in the studied domain and is inflexible for transfer learning.

Recently, deep learning has emerged as an effective method for analyzing CT images for lung nodule detection. Different from hand engineered feature-based CADe system, deep learning utilizes its self-learning power to automatically extract features from the input image [8]. Shin et al. [9] exploited the factors on deep convolutional neural networks (CNNs) architecture, dataset characteristics, and transfer learning by evaluating the performance of CNN on two CADe applications: thoraco-abdominal lymph node detection and interstitial lung disease classification. Setio et al. [10] proposed a CADe system for pulmonary nodules using multi-view convolutional networks (ConvNets), in which the discriminative features for nodule classification were automatically learnt from the training data. 3D CNNs for FP reduction via encoding multilevel contextual information in CADe of pulmonary nodules has been reported [11, 12]. Gruetzemacher et al. [13] further proposed 3D deep learning for both INC generation and FP reduction. Jiang et al. [14] introduced an effective CADe scheme for lung nodule detection based on multigroup patches, which were cut out from the CT images and enhanced by the Frangi filter. Kim et al. [15] proposed a multi-scale gradual integration CNN such that the feature representations of lung nodules were learned from multi-scale inputs with a gradual feature extraction strategy. Research work on integrating traditional features into deep learning based models to further improve the performance of detection and diagnosis of pulmonary nodules has also been reported [16–18].

Currently, most common deep leaning methods for CADe system take patches of the nodule CT images as inputs. For patch-based inputs, it is essential to select a proper input size for all nodules. In general, we need select the smallest input size that could include the largest region of interest (ROI) of the nodule candidates in the dataset. However, this will result in small nodules being a tiny fraction in the patch. Considering such problem, in this paper, we propose a study to project the CT image to the sinogram domain and explore additional effective features of nodules from the sinogram domain. Different from commonly developed CADe system which is designed to learn features using CT images, we propose a novel CNN-based CADe system directly applied in the sinogram domain to perform a self-directed learning of the effective features of lung nodules.

The remainder of this paper is organized as follows. Section 2 describes details of the proposed CADe system. Section 3 reports our experiment design and evaluation results of the proposed CADe system using the largest publicly available database built by the Lung Image Database Consortium and Image Database Resource Initiative (LIDC–IDRI). Finally, discussion and conclusions of our work, as well as future studies are given in Sections 4.

## Methods

Our proposed CADe scheme for pulmonary nodules in CT images contains two steps, INCs detection and FP reduction by sinogram-based nodule classification. Given a CT scan, the first step is to locate suspicious nodule candidates in order to narrow down detection scope. The next stage is to classify the identified INCs from the previous step. In the rest of this section, we will introduce these two steps in details accordingly.

### INCs detection

Given a CT scan, the task of the first step is to reduce the inspection area from the whole CT scan to a small number of suspicious areas, which were named as INCs. A principle for this task is to achieve a sensitivity of nodule detection to be close to 100% while keeping FP rate as low as possible.

To accomplish this task, we adopted our previously proposed hierarchical vector quantization (VQ) scheme to achieve a fast and adaptive detection of initial candidates of pulmonary nodules [6]. Different from the commonly used thresholding method [19], we first applied a high level VQ method for an accurate extraction of the lung volume. In this work, the first-order 3D neighbors were chosen for constructing a local intensity vector with seven elements. Through Karhunen-Loeve transformation [20], we selected the first few principal components that summed up at least 95% of the total variance for optimizing and reducing the dimensions of the feature vectors via the principal component analysis [21]. Then we applied a self-adaptive online VQ algorithm to these feature vectors for classification the lung volume. The proposed VQ algorithm is more robust to image noise comparing with the thresholding method. For the chest body volume, we classified it into two classes, where the lung volume was corresponding to the low-intensity class. Then several additional operations were applied to refine the extracted lung volume: (1) flood-fill operation [22]: fill the holes inside the extracted lung mask; and (2) morphological closing operation [23]: close the boundary on the parenchyma wall for juxta-pleural nodules.

**Fig. 1:**
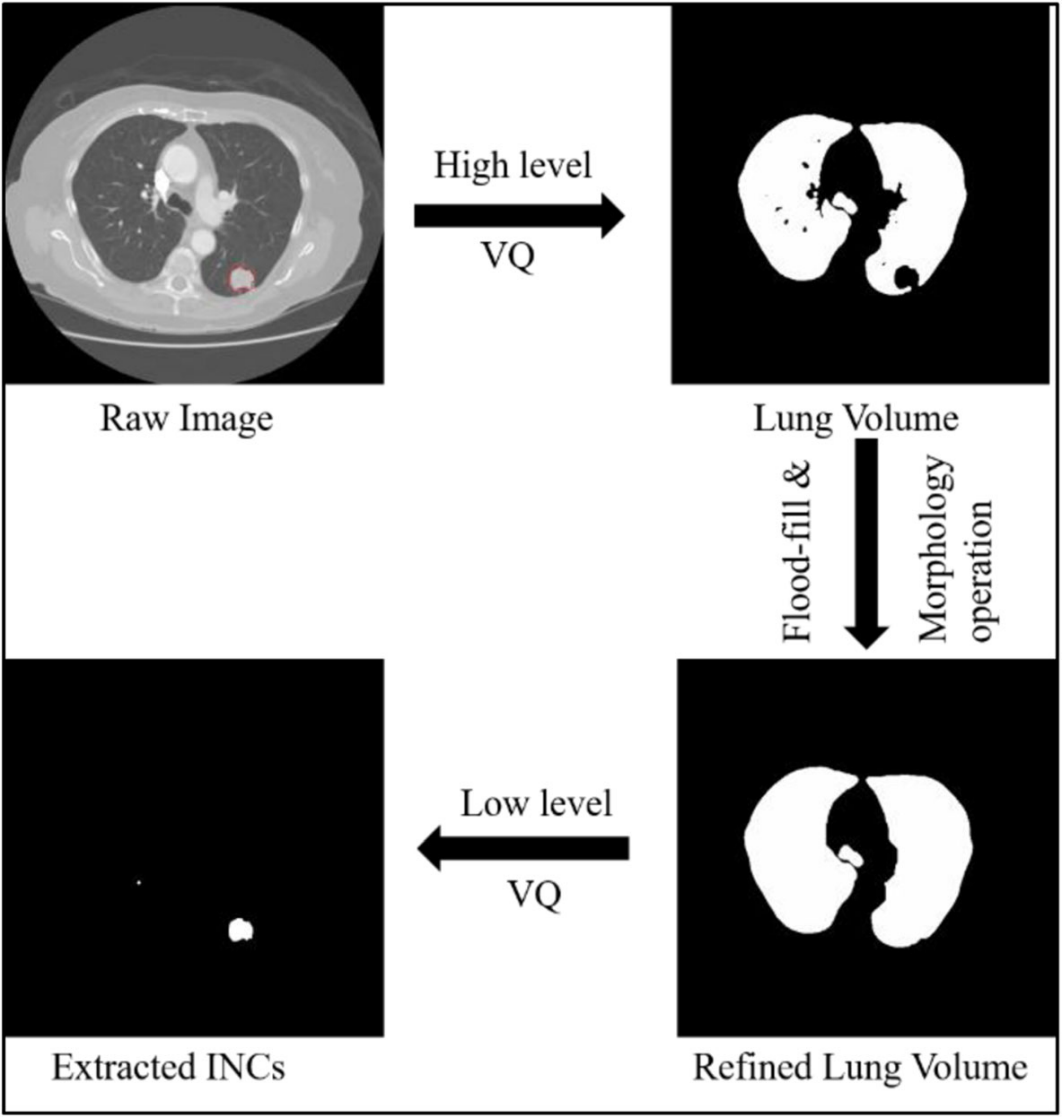
Examples of the initial nodule candidates detection

Then, a more diversified low level VQ was employed to detect and segment INCs inside of the lung volume. As we pointed out before, the task of detection of INCs requires our algorithm to be able to accurately classify all suspected nodules with sensitivity as close to 100% and a low rate of FPs. In order to achieve this task, we first studied the image intensity distribution inside of the lung volume and observed four class Gaussian mixtures corresponding to the low-intensity parenchyma, the high-intensity parenchyma, blood vessels, and INCs. Therefore, we set the maximum class number as four for performing the VQ algorithm which yielded the best segmentation results for INCs detection. An example of the INCs detection procedure was demonstrated in Fig. 1. The contour provided by LIDC database (red in Fig. 1) serves as the ground truth of locations for each nodule and will be used as the label for following FPs reduction.

### FP reduction by sinogram-based nodule classification

After acquiring the INCs, the next step is to further remove FPs. This can be achieved by nodule classification. One key problem for patch-based classification is to select a proper input size. One straightforward way is to select the smallest input size that could include the largest ROI of nodules in the dataset. However, it will result in small nodules being too subtle in the patch to be detected. Tan et al. [18] proposed a proportional patch extraction method, in which each ROI is centered using ration-cut and has the same object/background ratio as a patch size. That is, each patch is resized to the same size. However, this adaptive ratio-cut method still encounters a problem of missing nodule size information, which is very important in nodule classification. In this work, we propose to analyze the ROI of each patch in the sinogram domain via Randon transform [24] so as to unify input size with little information loss. Raw sinogram data contains multiple “projections” of the object being scanned, which are the Radon transformation of the structure of the object and contain additional rich information. Facing the challenges of limited research in this concept and unknown effective features in the sinogram domain, we design a CNN model using its automated feature learning power to explore effective sinogram features.

After a ROI of a nodule is located, we first convert it to the sinogram domain via Randon transform. Let ƒ(x, y) be a continuous function on R^2^ (two-dimensional space). The Radon transform is an integral transform defined by the line integral as shown in Eq. (1).

1 $$ {Rd}_f\left(\phi, s\right)={\int}_{L\left(\phi, s\right)}f\left(\mathrm{x},\mathrm{y}\right) dl $$

where L(*ϕ*,*s*) = {(x, y)∈ R^2^: xcosϕ + ysinϕ = *s*}and *ϕ* is the projection angle.

**Fig. 2:**
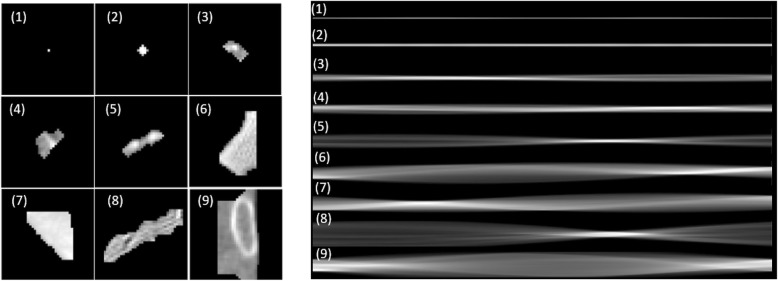
Examples of the nine typical initial nodule candidates in image domain (left) and sinogram domain (right)

Sinogram has two dimensions, representing number of bins and number of views. The number of bins is decided by the size of detector, while views are decided by the angle per each move. In this work, we designed the bin number to be 40 and modified different view numbers for performance comparison. The detected INCs are first centered and then resized to 40 × 40. The INCs centering and isolating follows the method proposed in ref. [14]. Based on each INC mask detected by VQ, we first locate each ROI and make sure it in the center patch. Then we remove all the surroundings based on the mask and resize each ROI to 40 × 40. Here 40 is one tradeoff between large and small nodule candidates. Since the CT ROI of size 40 × 40, the bin number of sinogram is chosen as 40. The view number is scanned from 40 to 640 to explore the effect of view numbers. Examples of the INCs in CT image domain and sinogram domain are shown in Fig. 2 with bin to be 40 and view number to be 640. Comparing INCs with different sizes and shapes by its CT and sinogram, we observed that sinogram contains shape and size information about the INCs, which are two important indicators for nodule classification. To be noted, the sinogram is scaled down for display purpose. Each sinogram has the same height with the CT image, which is 40.

For CT projection, more views mean that each view covers a smaller angle so that the projection will contain more detailed information. On the other hand, too many views will augment input size with redundant information presenting in the neighbor projection views, making it hard for the detection system to extract features and patterns. Regarding this, in order to capture the detailed information with more views as well as minimize redundancy, we design to convert the single channel sinogram into multi-channels for overcoming input size and increasing contrast among locations. For example, we convert 40 × 640 (bins × views) to 40 × 40 × 16 (bins × views × channels) as input for multiple channel model, which considers the size of CT ROI as reference. One straightforward way for this converting is to cut the whole sinogram image into several equal sized patches (direct-cut). However, such design will result in low channel-wise relevancy because the same location on different channels has little relevancy. Inspired by the concept of sparse-view CT [25], we design an interleave-cut where each channel includes views by a step size of k. In this way, each channel is actually a “sparse-view” of the original scan. Each channel contains the original neighbor views that are descriptors of the same location with correlated information, thus increasing contrast among each channel.

## Results

### Experiment setting

The proposed CADe system was validated on 208 patient cases from the publicly available LIDC-IDRI database. Each case contains at least one juxta-pleural nodule located at the lung boundary. We extracted INCs from the original CT images and centered them to construct a patch size of 40 × 40 as introduced in the method section. Based on the CT INCs, we then obtained its sinogram as input for the proposed CADe model.

**Fig. 3:**
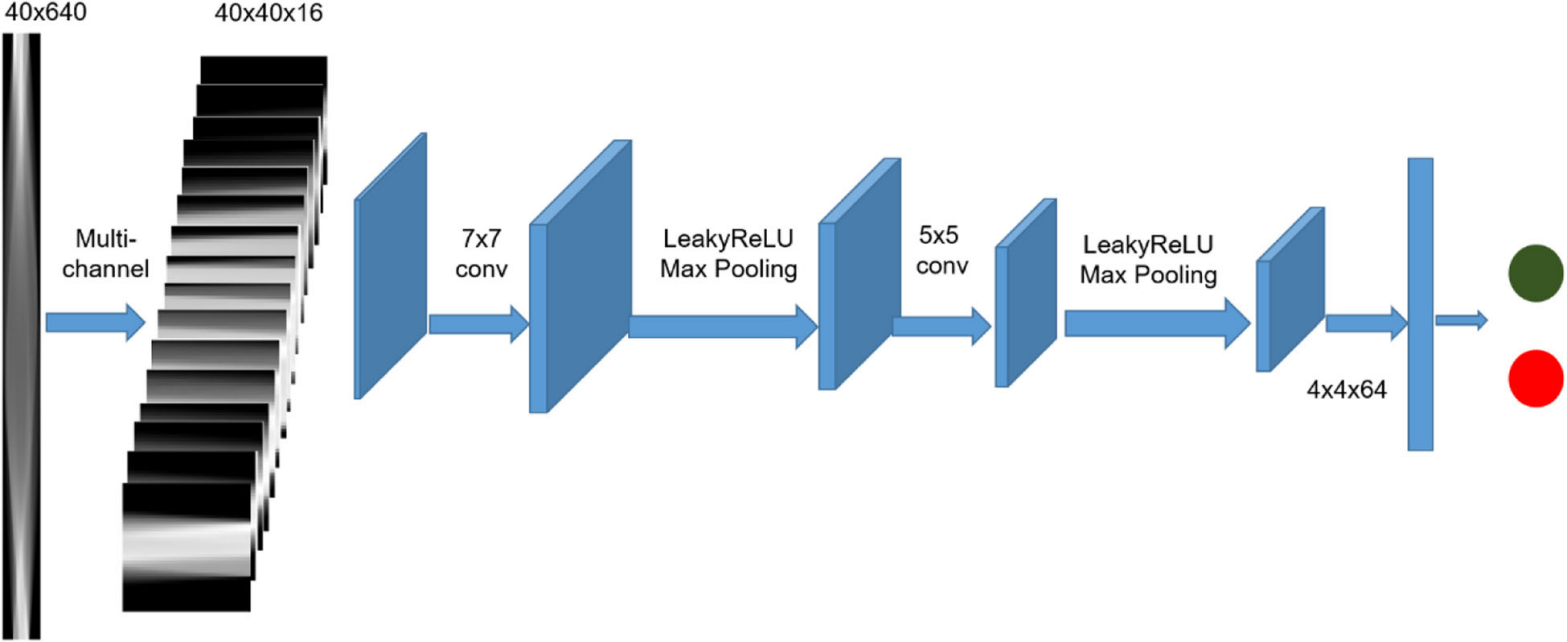
General workflow of our proposed sinogram based nodule detection method. The network contains 2 convolution layers with kernel sizes 7 × 7 and 5 × 5, with max-pooling layers following each convolution. Softmax is used as the final layer for generating risk probability

Figure 3 illustrates the general workflow of our proposed deep learning-based CADe system in sinogram domain. Here the sinogram size of 40 × 640 is used an example, which is converted into 16 channels as we introduced above. The workflow work was adapted to either multi-channel or single channel experiment as described below. In general, the network contains 2 convolution layers with kernel sizes 7 × 7 and 5 × 5, with max pooling layers following each convolution. Softmax is used as the final layer for generating risk probability. The network is trained with 25 iterations and batch size 30. Adam [26] is selected as optimizer with learning rate 1e-4, β1 = 0.9, β2 = 0.999. Early stop is adapted to avoid overfitting. We randomly split the dataset into 80% for training and 20% for testing. We split the dataset into training and testing in ROI level and make sure that same ROI will not belong to training and testing at the same time. We evaluated their nodule classification performances by the merit of area under the curve (AUC) of receiver operating characteristic.

### Comparison of the effect of projection view numbers

**Fig. 4:**
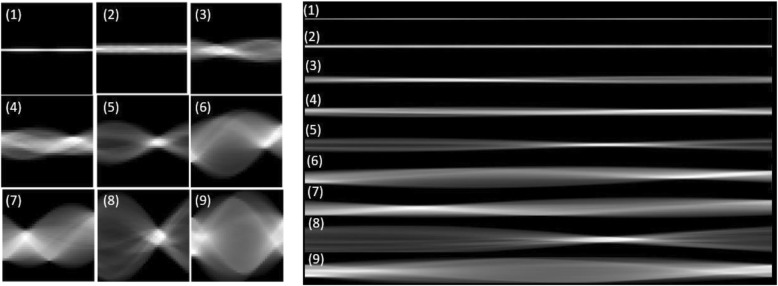
Examples of sinograms projected from the initial nodule candidates in Fig. 2 with different view numbers: 40 views (left) and 640 views (right)

We first conducted a comparison study to test the effect of projection view numbers on the performance of nodule classification. An example of the sinograms projected from the INCs in Fig. 2 with different view numbers: 40 views (left) and 640 views (right) are shown in Fig. 4. Both sinograms contains the structure and size information. However, the information details are different. More views may bring more information but also redundancy as we mentioned above. Therefore, we compared to the performance of sinograms with different views in this subsection. It is also noted that the sinogram of view 640 is scaled down for display purpose. Both sinograms of 40 views and 640 views have the same height with the CT image.

The settings for CNN model are listed in Table 1, which includes the kernel size, kernel numbers, activation function and so on for each layer. Table 2 shows the average values of AUC and its standard deviation with different projection view numbers. It showed that AUC value was increased as the number of projection view increased. That is, more views will provide more detailed information of the dataset as each view in such case covers a smaller angle of the projection. When the number of projection view equaled to 640, the system achieved a higher AUC value than that of other projection views. However, we also found that the AUC cannot always increase if we kept increasing view numbers due to too much redundancy.

**Table 1:** Convolutional neural network model settings for single channel input

Layer	Parameters
L1	Conv, 7 × 7, 32, LeakyReLU
L2	Maxpooling, 2 × 2, stride 2
L3	Conv, 5 × 5, 64, LeakyReLU
L4	Maxpooling, 2 × 2, stride 2
L5	Fully-Connected,1000, LeakyReLU
L6	Fully-Connected,2, Softmax

**Table 2:** Area under the curve values with different projection views

Models with different input	AUC (mean ± std)
Sinogram projection view 40	0.9048 ± 0.0007
Sinogram projection view 80	0.9104 ± 0.0005
Sinogram projection view 160	0.9109 ± 0.0003
Sinogram projection view 320	0.9113 ± 0.0004
Sinogram projection view 640	0.9121 ± 0.0001

### Comparison of different ways of setting multi-channel inputs

**Fig. 5:**
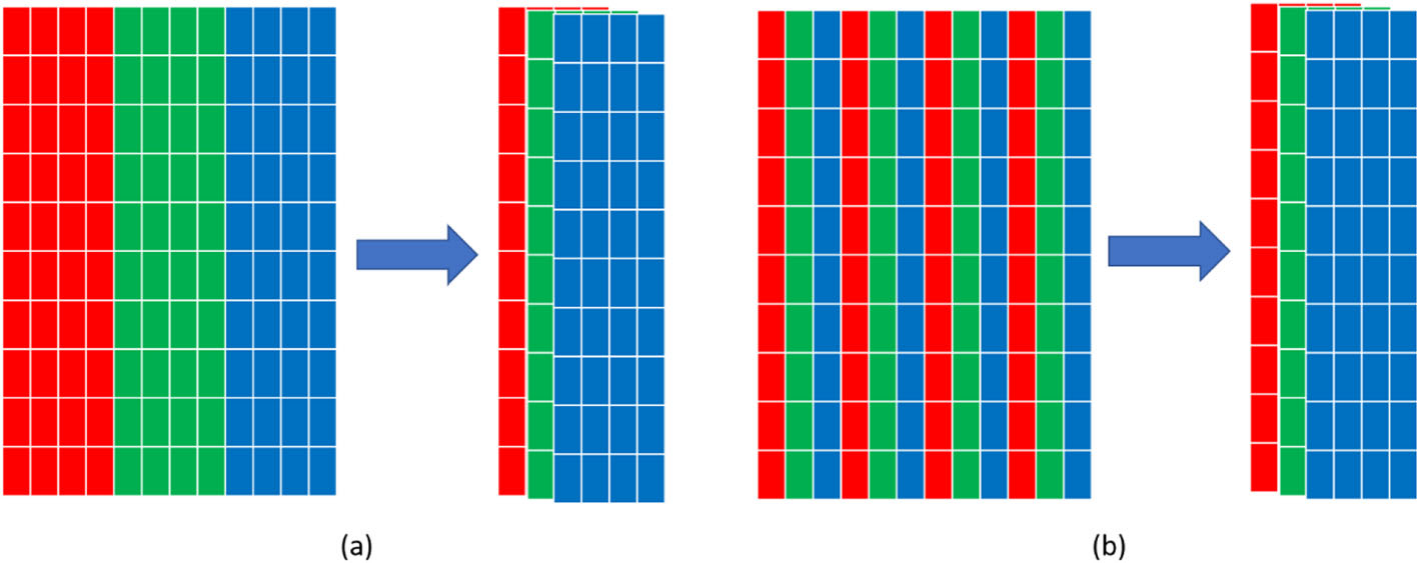
Illustration of direct cut (**a**) and interleave-cut (**b**)

**Fig. 6:**
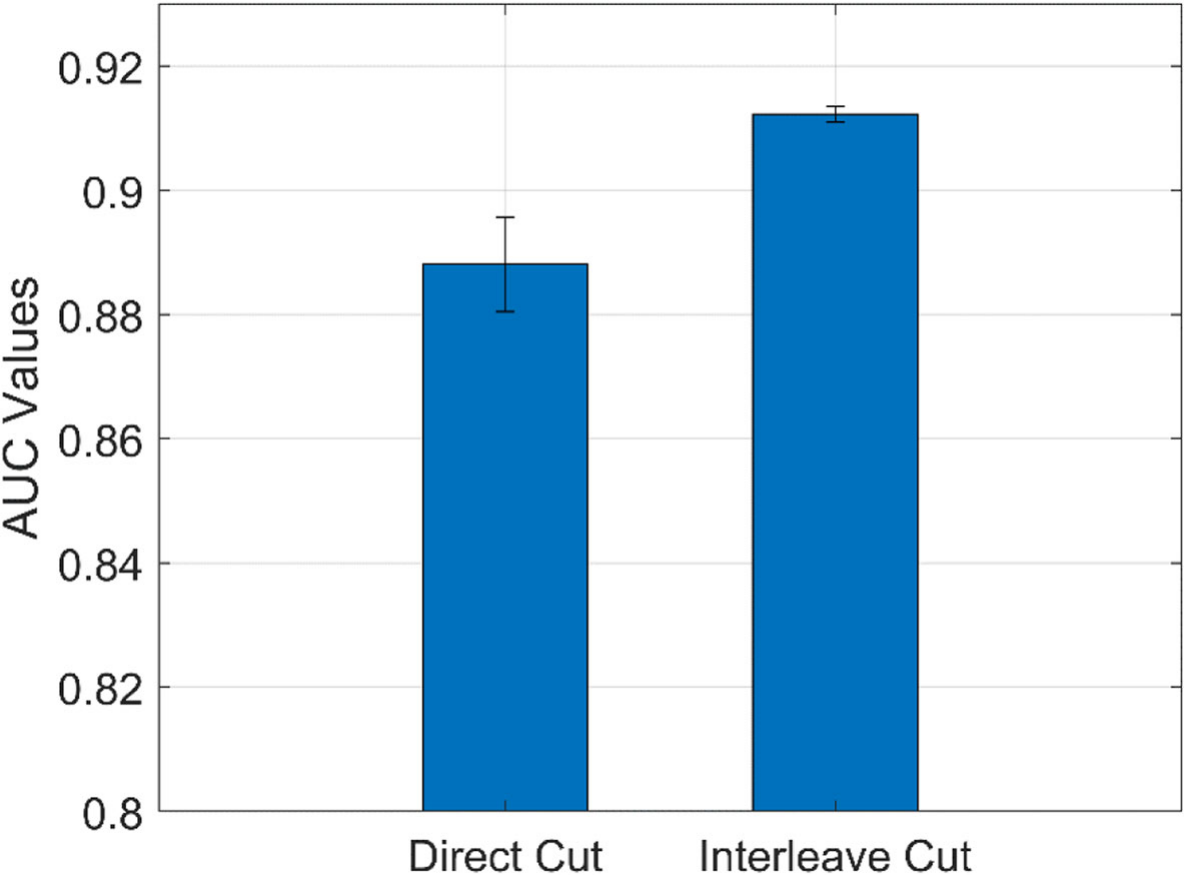
Area under the curve values of direct-cut and interleave-cut

From the above experiments, we can see the more projection view can bring us more information for achieving a high performance. However, too many views will augment input size with redundant information presenting in the neighbor projection views. In this study, we designed an experiment to convert the single channel sinogram into multi-channels for overcoming input size and increasing contrast among locations. We performed a comparison study of direct-cut and interleave-cut for setting multi-channel inputs. Figures 4 and 5 illustrate the methods of direct-cut and interleave-cut, respectively. Different from direct-cut, which cut the whole sinogram image into several equal sized patches with low channel-wise relevancy, we design an interleave-cut where each channel includes views by a step size of k. In this study, we set k equal to 3. As shown in Fig. 6, the interleave-cut achieved a higher AUC value comparing with that of the direct-cut.

### Comparison of performances via image domain and sinogram domain

**Fig. 7:**
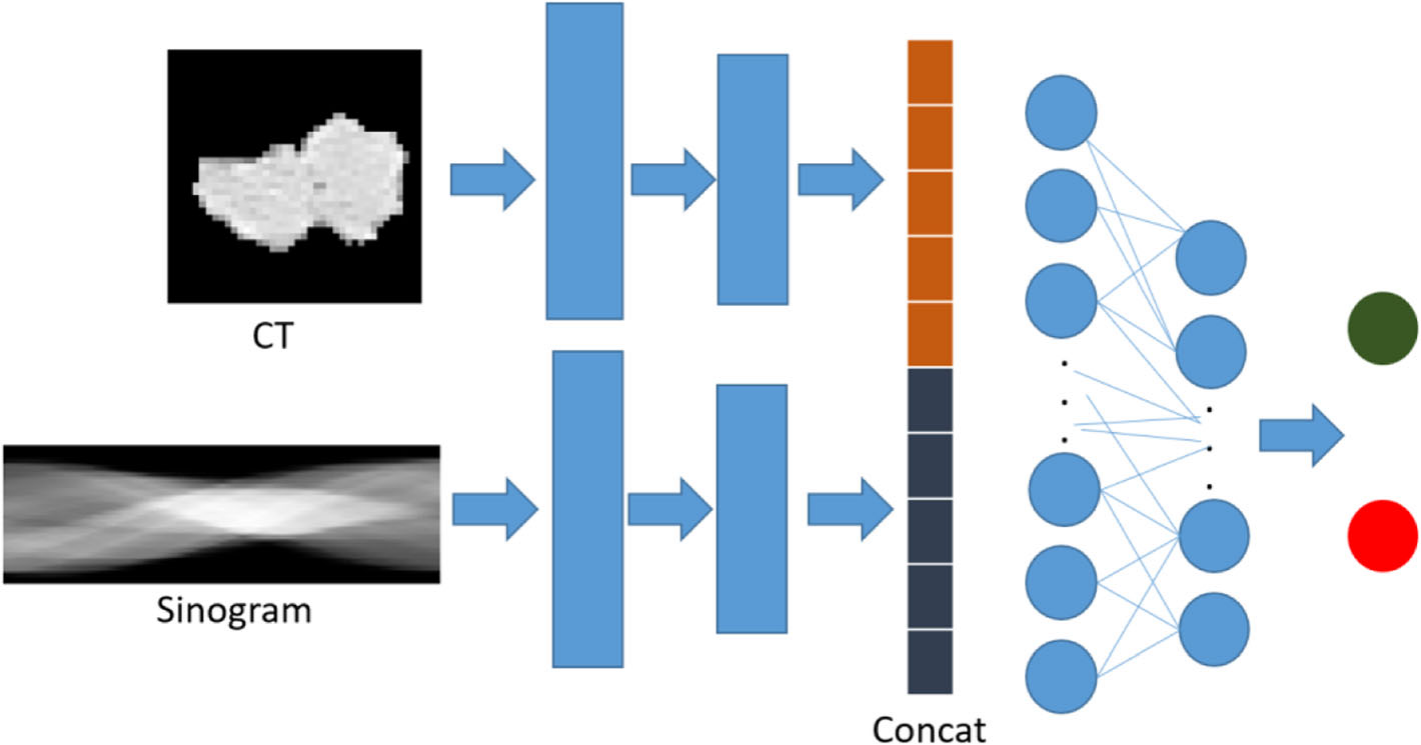
Workflow of our proposed scheme with combined inputs from image domain and sinogram domain

**Fig. 8:**
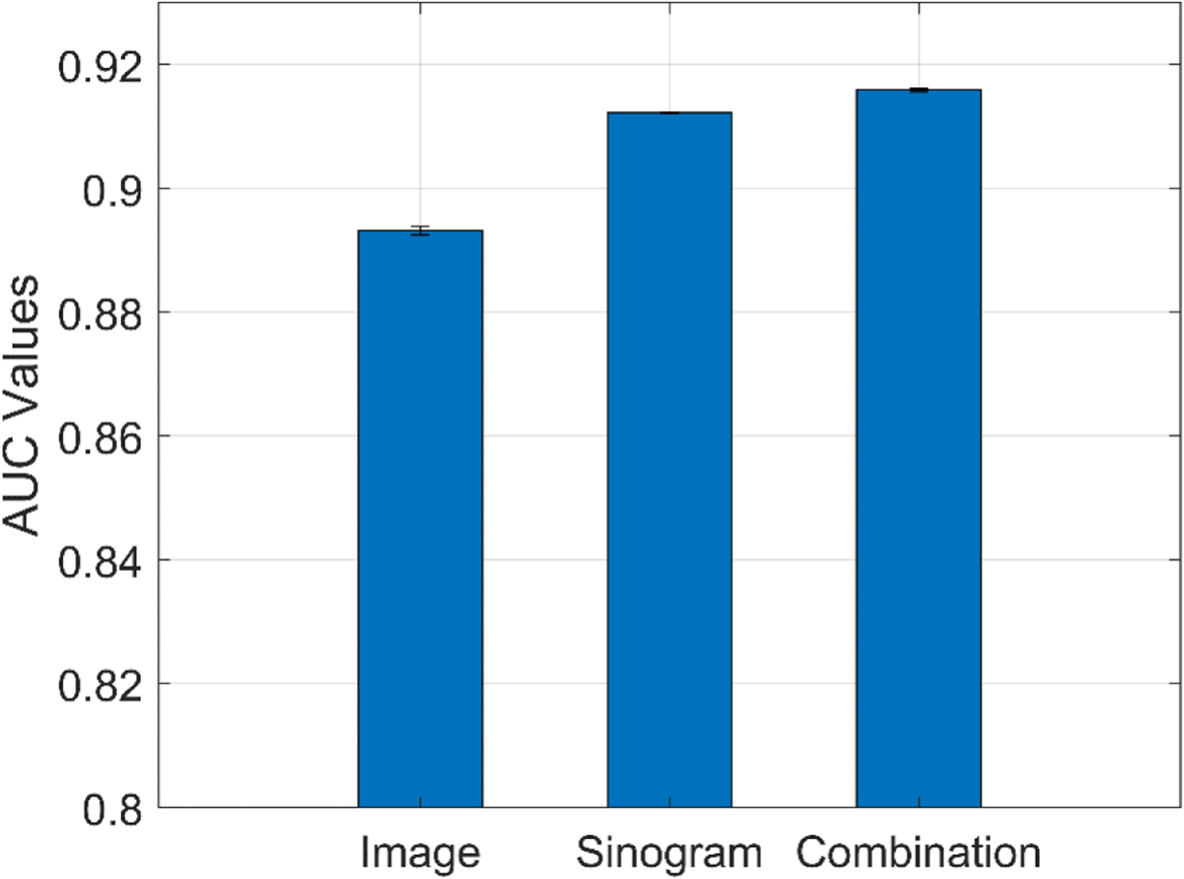
A comparison of performances via image patch, sinogram, and combination of both

We further conducted a comparison study of the performances of nodule classification via original nodule image patch, sinogram data, and combined inputs. The corresponding CNN model settings for single input and combined inputs are listed in Tables 1 and 3, respectively. Figure 7 shows that the workflow of our proposed scheme with combined inputs from image domain and sinogram domain. The number of projection views for sinogram is 640 in this experiment. As shown in Fig. 8, nodule classification in the sinogram domain is feasible and achieved an AUC value of 0.9113 which is higher than the classification performance using image patch only (with AUC value of 0.8933). When we combined inputs from both image domain and sinogram domain, the AUC achieved the highest value of 0.9154. This indicated that the sinogram domain provided supplemental information for nodule classification, thus improving the classification performance.

**Table 3:** Convolutional neural network model settings for combined inputs (sinogram and CT image)

Layer	Parameters
L1_1 (sinogram)	Conv, 7 × 7, 32, LeakyReLU
L2_1 (sinogram)	Maxpooling, 2 × 2, stride 2
L3_1 (sinogram)	Conv, 5 × 5, 64, LeakyReLU
L4_1 (sinogram)	Maxpooling, 2 × 2, stride 2
L1_2 (CT image)	Conv, 7 × 7, 64, LeakyReLU
L2_2 (CT image)	Maxpooling, 2 × 2, stride 2
L3_2 (CT image)	Conv, 5 × 5, 64, LeakyReLU
L4_2 (CT image)	Maxpooling, 2 × 2, stride 2
L5	Fully-Connected,1000, LeakyReLU
L6	Fully-Connected,2, Softmax

## Discussion and conclusions

In this paper, we proposed an improved CADe scheme for pulmonary nodule detection via deep learning in the sinogram domain. The proposed method can enhance our CADe framework by providing additional nodule information through different projection views in the sinogram domain, thus improving the detection performance. It can solve the different nodule size problem faced in the image patch-based CADe scheme. Experimental results demonstrated our method can improve the AUC from 0.89 to 0.91 from image domain to sinogram domain. Increasing projection views will also improve the performance. Moreover, a combination of sinogram and CT image could further improve the AUC to 0.92. This work has proven the feasibility of using deep learning-based nodule detection in the sinogram domain.

We evaluated our method on 208 patients from LIDC, with each case having at least one juxta-pleural nodule annotation. It is fact that the dataset is usually of relatively smaller size for medical imaging comparing to that in computer vision. Evaluation on a larger dataset is one of our future tasks to better assess the proposed model.

To the best of our knowledge, this is a pioneer work to perform CADe of pulmonary nodules in the sinogram domain. Sinogram, which is named because of its sine function alike visual representation, is the raw data obtained from CT scanner. Due to its insufficient for human interpretation, sinogram are usually transformed into CT image by tomographic reconstruction for human visual inspection, where information loss happens during reconstruction. This work demonstrated that deep learning can learn and extract additional effective features from sinogram domain, thus improving nodule detection. One possible resaon is that the sinogram can represent the shape, size or texture information of nodule candidates. The shape and size information can clearly be observed in Fig. 2. More research work is needed and under the way to interpret nodules in sinogram domain. This concept can be extended to other CT-based applications for detection and diagnosis. Further research on analyzing hand-engineered features in the sinogram domain and infusing those extracted features into the deep learning-based CADe scheme is under progress. Studies on the raw sinogram data from the detector is also one of our future research interests to advance the development of end to end CADe system. Additionally, including the surrounding tissues, i.e., the environment information could be another way to further improve the performance.

## Data Availability

The datasets used or analyzed during current study are public available.

## Abbreviations

CADe: Computer aided detection
CT: Computed tomography
CNN: Convolutional neural network
LIDC: Lung image database consortium
AUC: Area under the curve
ROC: Receiver operating characteristic
3D: 3 dimensional
ROI: Region of interest
LIDC-IDRI: Lung image database consortium and image database resource initiative
INCs: Initial nodule candidates
FP: False positive
VQ: Vector quantization
